# The essentialism of early modern psychiatric nosology

**DOI:** 10.1007/s40656-023-00562-x

**Published:** 2023-03-22

**Authors:** Hein van den Berg

**Affiliations:** Department of Philosophy, Institute for Logic, Language and Computation, , University of Amsterdam, Oude Turfmarkt 143, Postbus 94201 , 1090 GE Amsterdam, The Netherlands

**Keywords:** Essentialism, (Psychiatric) nosology, History of the concept of mental disorder, Christian Wolff, Sauvages, Cullen

## Abstract

Are psychiatric disorders natural kinds? This question has received a lot of attention within present-day philosophy of psychiatry, where many authors debate the ontology and nature of mental disorders. Similarly, historians of psychiatry, dating back to Foucault, have debated whether psychiatric researchers conceived of mental disorders as natural kinds or not. However, historians of psychiatry have paid little to no attention to the influence of (a) theories within *logic*, and (b) theories within *metaphysics* on psychiatric accounts of proper method, and on accounts of the nature and classification of mental disorders. Historically, however, logic and metaphysics have extensively shaped methods and interpretations of classifications in the natural sciences. This paper corrects this lacuna in the history of psychiatry, and demonstrates that theories within logic and metaphysics, articulated by Christian Wolff (1679–1754), have significantly shaped the conception of medical method and (psychiatric) nosology of the influential nosologist Boissier De Sauvages (1706–1767). After treating Sauvages, I discuss the method of the influential nosologist William Cullen (1710–1790), and demonstrate the continuity between the classificatory methods of Sauvages and Cullen. I show that both Sauvages and Cullen were essentialists concerning medical diseases in general and psychiatric disorders in particular, contributing to the history of conceptions of the ontology and nature of mental disorders.

## Introduction

Are psychiatric disorders natural kinds? In his early work (Hacking 1991), which he has later revised (2007), the philosopher Ian Hacking argued that the human sciences study what he calls ‘human kinds’, which are not natural kinds. Rachel Cooper (2004) criticizes Hacking’s argument, whereas Cooper (2014) contains extensive discussion of whether mental disorders are natural kinds or not. Adriaens and De Block (2013) provide an account of why we essentialize mental disorders, arguing that even in present-day psychiatry essentialism is a pervasive problem. In philosophy of psychiatry and psychiatry, debates about natural kinds are also prevalent. For example, Kendler et al. (2011) reject essentialism about mental disorders because they are causally heterogeneous and messy, but argue that mechanistic property cluster kinds are useful for capturing the nature of mental disorders. Historians of psychiatry have also debated essentialism and whether mental disorders have been conceived of as natural kinds, most famously Foucault in *The History of Madness* (2006), with a focus on eighteenth-century nosology (see also Berrios, 2012). The history of Kraepelin’s account of mental disorder is discussed in relation to natural kinds in (Engstrom & Kendler (2015); Kendler & Jablensky (2011)), whereas debates about the history of nosology in psychiatry, including the history of the *DSM*, are debated in (Kendler & Parnas 2012 and Horwitz, 2021).

Although historians of psychiatry are sensitive to general philosophical influences on the development of psychiatry, they have paid little attention to the specific impact of (i) theories within *logic*, and (ii) theories within *metaphysics* on theories of psychiatric science and method, psychiatric disorders, and psychiatric classification. However, historically, *logic* and *metaphysics* have significantly influenced theories of scientific method and classification in the sciences. Thus, Ereshefsky (2001) argues that essentialist theories of biological classification can be traced back to the *metaphysics* of Aristotle, according to which objects have real essences. Moreover, these metaphysical theories were related to influential Aristotelian *logical* theories, according to which definitions provide us with knowledge of real essences of a kind (Ereshefsky, 2001).

This paper provides a novel synthesis of the disciplines of the history of logic, history of metaphysics, and the history of psychiatry. I argue that early eighteenth-century theories within logic and metaphysics, articulated by Christian Wolff (1679–1754), have significantly shaped the (psychiatric) nosology of the influential nosologist Boissier De Sauvages (1706–1767). More specifically, I argue that Wolff’s logic and metaphysics shaped Sauvages’ conception of (a) medical science and method, (b) the nature of medical disorders in general and psychiatric disorders in particular, and (c) conceptions of (psychiatric) classification. After treating Sauvages, I will discuss the method and little known essentialist views of the influential nosologist William Cullen (1710–1790). This demonstrates the similarity between the classificatory methods of Sauvages and Cullen. Although non-essentialist views on diseases were not a real option for medical researchers in the eighteenth century, I provide a novel contribution to the history of medicine and psychiatry by investigating the logical and metaphysical origin, nature and the logical and metaphysical presuppositions of eighteenth-century (psychiatric) essentialism.

The work of Sauvages has been studied by King (1966), who notes the influence of Christian Wolff on Sauvages. Martin (1990) provides an account of the scientific context within which Sauvages was trained and worked. French (1990) describes Sauvages’ work in relation to Stahl and Hoffman, and Williams (2003) discusses Sauvages within the context of providing a history of medical vitalism in enlightenment Montpellier, also discussing his nosology. Finally, Huneman (2008) discusses Sauvages within the context of an account of Montpellier vitalism and its influence on the emergence of alienism in France, whereas Foucault (2006) provides brief discussion of Sauvages (and Cullen).

Cullen remains little studied. Risse (1974) describes Cullen’s letters and consultation practice. Cullen’s views on melancholia are discussed by Jackson (1983). Barfoot (1993) provides an account of philosophy and method in Cullen’s medical teaching, whereas Bynum (1993) discusses Cullen and the nervous system. Kendell (1993) briefly discusses Cullen’s nosology. Finally, Dyde (2015) reconstructs the meaning of neurosis in Cullen’s work, and Beatty (2016) discusses Cullen in her history of the concept of nervous disease. The essentialism of Cullen has received little to no discussion to the best of my knowledge.

Although the influence of Wolff on Sauvages has been noted by King (1966), King does not discuss the content of Wolff’s logic, his theory of axiomatic science, and his metaphysics. These topics, and their impact on Sauvages, will be the focus of this paper. In this way, I provide a novel account of the impact of logic and metaphysics on eighteenth-century (psychiatric) nosology. Martin (1990) briefly notes Sauvages’ essentialism, but the nature of this essentialism and the logical and metaphysical Wolffian context within which this essentialism is to be interpreted are nowhere discussed by Martin. This will be the task of this paper.

Finally, it is important to describe why I focus on (i) the nosologies of Sauvages’ and Cullen and (ii) their views on psychiatric disorders, given that they wrote nosologies on all diseases in general. As to (i), I focus on Sauvages because, as Foucault (2006) and in more detail Huneman (2008) have shown, Sauvages was an influential medical researcher who significantly influenced the rise and birth of psychiatry around 1800. Moreover, because Sauvages’ work contains much explicit philosophical reflection on nosology, nosological method, and medical method in general, he is a suitable figure to demonstrate the influence of eighteenth-century theories within logic and scientific methodology on (psychiatric) nosology, which is the core contribution of this paper. I focus on Cullen because Cullen was a nosologist and medic who was influential at the end of the eighteenth century, and shaped, mainly through his teaching and textbooks, the views of many of his students and medics (Doig et al., 1993). Cullen is also discussed because, even though he was based in Edinburgh and exposed to different philosophical currents then Sauvages, there is a great deal of continuity between the views of Sauvages and Cullen, including in their adoption of a causal method of classification. One aim of this paper is to demonstrate this continuity and thus to highlight the impact of certain common methods of classification in eighteenth-century medicine.

As to (ii), note that I will describe the general method of classification adopted by Sauvages and Cullen, a method adopted for both psychiatric and non-psychiatric disorders. Thus, I will discuss their views on nosology in general, which are essentialist. It is important to point out this essentialism for diseases in general because it is very rarely discussed. I subsequently focus on their views on psychiatric disorders, because Sauvages’ and Cullen’s views on the nature and classification of mental disorder, and especially their essentialism concerning psychiatric disorders, are also very little discussed despite the importance of these authors for the birth of psychiatry. By discussing these essentialist views on mental disorder, this paper contributes to the history of the ontological conceptions of mental disorder. Through our discussion of eighteenth-century conceptions of mental disorder, we will also see that in the eighteenth century some researchers such as Sauvages thought that mental disorders have multi-factorial causes. However, Sauvages remained committed to essentialism. This is philosophically interesting, insofar as some contemporary philosophers of psychiatry (Kendler et al., 2011) take the fact that mental disorders are multi-factorial diseases (in contrast to mendelian diseases) to be a reason to reject essentialism. If history is a guide, it seems possible to combine essentialism with the fact that mental disorders have many causes.

In Sect. 2, I provide a description, following Ereshefsky (2001), of core features of essentialist classifications. I argue that these features were widespread throughout history in general and influenced eighteenth-century nosologists in particular. In Sect. 3, I analyze Wolff’s and Sauvages’ shared conception of science, Wolff’s logic, theory of division, and conception of essence, and Sauvages’ views on nosology and the causes of psychiatric disorders. I argue that Sauvages was an essentialist and that he aimed, following Wolff, to give real definitions of (psychiatric) disorders or diseases. In Sect. 4, I analyze Cullen’s philosophy of classification and his views on the causes of psychiatric disorders. I argue for continuity between the methods of Sauvages and Cullen and for the fact that both were essentialists.

## A brief description of essentialism in the eighteenth century

In this section, I will describe some core features of essentialist classifications, building on the work of Ereshefsky. Ereshefsky takes the eighteenth-century naturalist Linnaeus to be a paradigmatic example of an essentialist. However, this view has been challenged by (Müller-Wille, 2007). According to Müller-Wille (2007), which is a very rich article which I cannot explain in detail, Linnaeus did not classify by logical division but used inductive and empirical methods for providing classifications. I am completely convinced by the reading that Linnaeus adopted these inductive and empirical methods, but think that Linnaeus can still be called an essentialist on metaphysical grounds. This is because Linnaeus, as Müller-Wille agrees, distinguishes between artificial and natural classifications and argues that we can provide natural definitions of some classes. This suggests, to me, that Linnaeus still thinks there are natural kinds that carve nature at its joints, even if having knowledge of these kinds is difficult to obtain and requires empirical and inductive methods. It is certainly the case that the medical researchers who I discuss also adopt many empirical methods. However, my contention is that from a metaphysical perspective, which concerns the metaphysical interpretation of classes, they are still essentialists like I think Linnaeus is too.

Ereshefsky provides an account of essentialism that will be useful to use when analyzing the eighteenth-century views of Sauvages and Cullen, for these researchers conformed to this account of essentialism. Ereshefsky defines essentialism as follows:

According to Essentialism, each entity has an essential feature that makes it the type of entity that it is. That feature is an entity’s real essence. The real essence of an entity occurs in all and only entities of that type, and it helps us understand why entities of that type do the sorts of things they do. For the essentialist, real essences capture the fundamental structure of the world; or to use Plato’s phrase, they “carve nature at its joints”. (Ereshefsky, 2001, p. 17)

Ereshefsky further notes that members of a kind share common necessary properties, which are caused by the real essence of an entity (2001, p. 17). Such necessary properties are required for membership in a kind. For example, the real essence of gold causes pieces of gold to have the necessary properties “of being soluble in certain types of acids, reflecting certain wavelengths of light, and having a particular range of malleability” (2001, p. 17). If someone knows the real essence of an object, she can explain why the object has the necessary properties it has. Note that this is a completely metaphysical account of essentialism, which I think is not affected by Müller-Wille’s (2007) argument, which mainly concerns the empirical and inductive methods of eighteenth-century naturalists.

According to Ereshefsky, essentialist scientific classification in the eighteenth century followed the Aristotelian method of logical division (Ereshefsky, 2001, p. 201). Here, Müller-Wille’s argument that Linnaeus’ did not follow the method of logical division is important. However, I will argue in what follows that medical researchers like Sauvages and Cullen did follow this method. Moreover, as we shall see below, the method of logical division again largely consists of metaphysical claims or metaphysical interpretations of logical categories, which I think one can adopt even if one agrees with Müller-Willle (2007) that eighteenth-century naturalists adopted inductive methods.

The method of logical division, as described by Ereshefsky, postulated five predicables: a definition, a genus, a differentia, a property and an accident (Ereshefsky, 2001, p. 201). Definitions describe which characteristics pertain to an object in virtue of which it belongs to a particular *kind*. Moreover, definitions provide us with the real essence of the members of a kind (2001, p. 201). Definitions are given by the traditional Aristotelian method of providing a genus and a differentia. Thus, for example, the concept of “man” is traditionally defined as a “rational” (differentia) “animal” (genus). Properties are characteristics that follow from an object’s essence and are found in all the members of a particular kind (2001, p. 201). To return to our example: “animal” is part of the essence of “man”. From the fact that “man” is an “animal”, it also follows that “man” is a “substance”, insofar as the concept of “substance” is contained in the concept of “animal” (all animals are substances). Hence, “substance” is a property of “man”. Accidents are accidental properties and have no relation to the essence. Thus, for example, insofar as some man are pale, “paleness” is an accidental property of man. Species are distinguished from other species of a genus by their differentia. Thus, “man” is a species of “animal”. The essence of a species is given by its definition (2001, p. 202). Followers of the method of logical division also distinguish between necessary properties and accidental properties of individual objects.

In the next sections, we will see that the method of logical division (as described above) was adopted by the famous eighteenth-century philosopher Christian Wolff, who, as we shall see, significantly influenced the classificatory practices of Sauvages.

## Wolffian logic and the nosology of Sauvages

François Boissier de Sauvages (1706–1767) is famous for introducing a nosology for diseases in the spirit of Thomas Sydenham. As Martin (1990, p. 111) notes, Sauvages (1706–1767) is known as a celebrated Montpellier medical professor, who wrote an influential classification of diseases. Martin wants to describe the influence of Bacon, botanical method, and Newtonian physics on the work of Sauvages (ibid.). It is common to describe the affinity between Linnaeus and Sauvages and the similarity between their classificatory practices. Thus, as Munsche and Whitaker (2012) explain, Sauvages’ published the first version of *Nosologie méthodique* in 1731, when Linnaeus was a medical student. Linnaeus used this work for his own *Genera Morborum* of 1759, in which he classified diseases. According to Munsche and Whitakker (2012), these two authors influenced each other’s subsequent works, a fact which demonstrates the influence of Sauvages on medical classifications in the eighteenth century. The interaction between Linnaeus and Sauvages also illustrates the mutual influence of natural history and medical nosology upon each other. Martin (1990) describes the influence of the botanical methods of Tournefort on Sauvages’ work. Tournefort classified plants in accordance with their *essential character,* i.e., the plant's reproductive parts (1990, p. 119). According to Martin, Sauvages adopted a form of essentialism from Tournefort: following Tournefort, Sauvages wanted to discern the “essential characteristics of species of diseases” (1990, pp. 125–126). In a classic paper about Sauvages, King (1966) remarks that Sauvages was influenced by Christian Wolff. According to King, commenting on the *Pathologia methodica* (1752), Sauvages, under the influence of Wolff, “touches upon numerous aspects of logic and relies upon definitions and their implications” (King, 1966, p. 47). In addition, Sauvages copied Wolff’s distinction between historical, philosophical, and mathematical knowledge, Wolff’s distinction between *principium* and *causa*, and Wolff’s distinction between mechanical and physical principles (1966, pp. 48–49).

In this section I want to further develop the historical study of Wolff’s influence on Sauvages, with the goal of demonstrating that Wolff’s logic, conception of definitions, and conception of proper science, which King does not discuss, significantly shaped Sauvages’ conception of medical science and disease. In addition, Wolff, as we will see, adopted a traditional essentialist position according to which objects are a type of entity in virtue of their essence. Wolff further adopted the traditional Aristotelian theory of logical division, which we have already discussed, and argued that species and genera reflect the essences of nature. Sauvages also adopted these positions. In order to demonstrate Wolff’s influence on Sauvages, I will first consider both these authors' views on proper science. Through studying this topic, we will see that there is enormous continuity between the methodological and logical views of Wolff and Sauvages. On this basis, we can subsequently argue more convincingly that there is also enormous continuity between the logical and metaphysical views of Wolff and Sauvages that concern essentialism, namely a continuity between their views on logical division, which was traditionally a topic that pertained to metaphysics and logic, and essences.

### Wolff’s and Sauvages’ conception of proper science

As van den Berg and Demarest (2020) have argued, Wolff accepts a variety of a traditional axiomatic ideal of science, which has been modeled by de Jong and Betti’s ‘classical model of science’ (de Jong & Betti, 2010. See also van den Berg, 2020). According to this ideal, a proper science has fundamental concepts and non-fundamental concepts are defined in terms of these fundamental concepts. In addition, a proper science has fundamental propositions and non-fundamental propositions are grounded by or demonstrated from these fundamental propositions (de Jong & Betti, 2010). The propositions of a proper science should also be certain, i.e., known to be true. Wolff himself argued that any proper science should follow what he called the mathematical method (see for descriptions of this method Blok, 2016, pp. 13–45; Shabel, 2003, pp. 49–52; Dunlop, 2013; Gava, 2018, pp. 279–284). As van den berg (2021, p. 272) explains, Wolff’s mathematical method moves from definitions to axioms, and from axioms to theorems and problems. Axioms, which are either axiomata (which show that something is the case) or postulata (which show that something can be done or constructed), are derived from definitions. Theorems are then derived from axioms (axiomata and postulata) through strict syllogistic demonstrations (Wolff, 1999 [1750]). See for a quantitative study of the spread of Wolff’s mathematical method the as yet unpublished van den Berg, Parisi, Oortwijn, and Betti “The Spread of the Mathematical Method in Eighteenth-Century Germany: A Data-Driven Investigation”).

Next to arguing that all sciences should have a strict axiomatic structure, Wolff also had strict views on the hierarchy of sciences (see on this topic van den Berg, 2013, on which I draw in the following). According to Wolff, sciences are ordered from higher to lower and the so-called higher or preceding sciences provide concepts and propositions which can be used in proofs of the lower sciences. For example, Wolff argues that the higher science of ontology grounds the lower sciences of psychology and physics.

Such general notions are the notions of essence, existence, attributes, modes, necessity, contingency, place, time, perfection, order, simplicity, composition, etc. These things are not explained properly in either psychology or physics because both of these sciences, as well as the other parts of philosophy, use these general notions and the principles derived from them. Hence, it is quite necessary that a special part of philosophy be designated to explain these notions and general principles, which are continually used in every science and art, and even in life itself, if it is to be rightly organized. Indeed, without ontology, philosophy cannot be developed according to the demonstrative method. (Wolff 1963 [1728], 40)

Wolff’s rationalistic conception of axiomatic science was combined with a form of empiricism in his account of the natural sciences. As van den Berg and Demarest (2020) have shown, Wolff “combines experimental research with a deductive mode of presentation” (2020, p. 385). Natural science should proceed in the demonstrative fashion and should provide strict syllogistic demonstrations. The premises of such demonstrations are “definitions based on experience and propositions of experience” (p. 385). Wolff thought that such empirical premises express *clear experiences*, and are thus certain (p. 386). Hence, Wolff harmonized the ideals of experimental science and axiomatic science.

Having discussed the basics of Wolff’s conception of science, we may now turn to the views on proper science and medicine of Sauvages. In his ‘Preliminary Discourse’ to *Methodological Nosology* (2015 [1772]), Sauvages remarks that medicine should not be based on unfounded hypotheses but on certain principles drawn from experience: “You should carefully discard all theory with precarious principles based on a whim rather than on repeated experience, and supported by possibilities, rather than on certain facts and experiences” (2015 [1772], p. 481). Medicine, according to Sauvages, should be based on such certain empirical principles, which he also calls, no doubt following Wolff, *Experiences incontestables* (p. 483). Hence, according to Sauvages, medicine should be a certain science based on incontestable and certain principles. The problem, however, is that medicine has not been furnished with such certain principles: “[…] Medicine, which is the noblest and the most ancient of all Arts, has made little progress, and its theory is unable to initiate Candidates to practice, only providing a few real and incontestable principles” (p. 482).

Apart from stating that medicine should be based on certain empirical principles, Sauvages also clearly accepts Wolff’s axiomatic or demonstrative method for medicine. As Sauvages puts the point:

One must not allow in Medicine any principles except those that are as certain as those that we acquire by the testimony of the senses. Now, following the method of the Geometrists, these principles are none other than the experiences and syllogisms deduced, one from the other. (2015 [1772], p. 483)

Here, Sauvages seems to construe medicine as an axiomatic science based on certain empirical principles from which non-fundamental propositions are deduced through syllogisms. Moreover, he explicitly states, like Wolff, that we should follow the method of the geometrists. This reading is strengthened by Sauvages’ definitions of proof and demonstrations, which all conform to Wolff’s definitions of these terms. Wolff defines a demonstration as a syllogism starting with definitions, clear experiences, or axioms (1742, p. 95). Similarly, Sauvages states:

If we use syllogisms to demonstrate a proposition by means of some others that are already known, this is called a *Proof* (une Preuve) and a *Demonstration* (une Démonstration), when we only use as premises *Definitions* (définitions), *Unquestionable experiences* (d’Expériences incontestables), *Axioms* (d’Axiomes), and *Propositions* (Propositions) already demonstrated. (2015 [1772], p. 483. Translation amended)

In addition to adopting Wolff’s account of the mathematical method and demonstration, Sauvages adopts a view on the hierarchy of sciences that is similar to that of Wolff. According to Sauvages, higher sciences, such as mechanics, provide concepts and principles for medicine in order to provide proofs in the latter science. As Sauvages puts the point:

Finally, Medicine has to borrow from philosophy, from Mechanics, from Geometry, and from other general sciences, not only terms but also principles; it is from these fields that the Physicians borrow the propositions demonstrated, and they are not required to demonstrate these propositions by themselves. (2015 [1772], p. 483)

Sauvages illustrates the hierarchy of sciences by explaining how hydraulics can be of use to physicians:

However, I say that anyone who does not study in Hydraulics the general property of fluids, and how to understand their speed and force, will be unable to draw from Geometry and Mechanics the knowledge of the capacity of the vessels, their diameters and surfaces, as well as the knowledge of the hardness of solids, the movement and tone of fibres. Such a person, I say, will never succeed in obtaining perfect knowledge of the animal economy, and will not acquire the theory of hearing and sight without studying Acoustics and Optics. (2015 [1772], p. 484)

Finally, within his account of the hierarchy of sciences, Sauvages stresses that physicians should adopt the demonstrative method, which as we have seen is Wolff’s axiomatic method. It is thus clear without a shadow of a doubt that Sauvages adopted his conception of proper science from Wolff. As Sauvages puts the point:

In so far as the Physicians ignore the demonstrative method, there will be no principle upon which practice can be built, and which has the certainty it demands; the theory of this Art will always be uncertain, and everyone will assert his opinion in proportion to the mind and the credit that he has. (2015 [1772], p. 483)

### Wolff’s logic and conception of essence.

Wolff adopts the Aristotelian theory of logical division that was first developed by Aristotle and that, as we have seen, is a core feature of essentialism. Wolff defines an essence as that which is constantly present in a thing and not derived or determined by something else (1728, p. 145. I will further explicate the concept of essence below). Attributes are those necessary properties that follow from the essence of a thing (1728, p.146). Modi are changeable things which are not determined by or related to the essence (1728, p. 147). Definitions are given by genus and specific difference (1728, p. 208), and allow us to distinguish kinds of objects from each other (1728, p. 190). Finally, Wolff distinguishes genera and species, and, as I will explain below, argues that species pick out essences in nature.

The theory of definition adopted by Wolff is traditional. In his *German Logic*, Wolff describes a definition (*Erklärung*) as a clear, distinct and exhaustive concept (1742, p. 44. See for Wolff’s account of definitions also van den Berg, 2014, pp. 19, 59–60). Such a concept, Wolff argues, is applicable to multiple things of a kind and enables us to differentiate particular types of objects from other objects (1742, p. 44). A concept is clear if it suffices to recognize the objects to which it applies (1742, p. 18). A concept is distinct if we can specify its marks, i.e., the partial concepts contained in it on the basis of which we know an object (1742, p. 20). Finally, a concept is exhaustive if its marks suffice to know an object and differentiate it from other objects (1742, p. 23).

Wolff distinguishes between nominal definitions and real definitions. Nominal definitions provide an account of properties on the basis of which an object can be distinguished from other objects. For example, if one defines the word clock by saying it is a machine (genus) which specifies the hours (differentia) one provides a nominal definition of the word clock (1742, p. 18). Real definitions show how a thing is possible, or they show how a thing is generated, and thus explicate the essence of composite objects (1742, p. 48, pp. 52–53). We know the essence of a composite object if we know its parts and the mode of composition of the parts (Wolff, 1738, p. 29). Thus, if one specifies the parts of a clock and their mode of composition one explicates the essence of a clock and provides a real definition of a clock (Wolff, 1742, p. 48). In a similar way, if one specifies the parts of the eye and their mode of composition, one provides a real definition of the eye and explicates its essence (1742, p. 53).

Wolff follows the traditional dictum that we provide a definition of a concept if we specify its genus and differentia (1728, p. 208). However, such definitions need not be real definitions. In my opinion, definitions in terms of genus and differentia would be classified as nominal definitions by Wolff. For a real definition, it is required that we explain how a thing is generated. This requires that we know what kinds of things are required for an object to be generated and what each of these things contributes to the generation of an object (1742, p. 54). In geometry, for example, real definitions are not provided by a definition in terms of genus and differentia. Thus, we can give a nominal definition of a circle as a round plane figure (genus) whose boundary is equidistant from a fixed center (differentia). However, we only provide a real definition of a circle by constructing it, i.e., by drawing a straight line around a fixed point, thus showing how it can be generated. Hence, Wolff claims that in geometry we assume points and lines and through their movement obtain real definitions of planes (1742, p. 55). In a similar fashion, real definitions in natural science show how an object is possible or generated.

Central to Wolff’s account of real definition is the notion of essence. In his *German Metaphysics*, Wolff defines essence as that which contains the ground for the properties of objects (1738, p. 18. See also van den Berg, 2014, p. 61). Thus, for example, if we know the essence of the eye we know *why* the eye has the capacity for sight. We understand the essence of an object if we understand how it is possible (1738, p. 19). According to Wolff, the essence of objects is necessary, eternal, and unchangeable (1738, pp. 20–21). As said above, the essence of composite things consists in the mode of composition of its parts (1738, p. 29). It follows, according to Wolff, that composite things are similar, i.e., belong to the same kind, if their mode of composition (their essence) is similar (1738, p. 29).

Wolff explicitly links the notion of species and genus to his conception of essence. He argues that insofar as objects share an essence, they belong to the same species (*Art*). (1738, pp. 95–96). The differentia of a species consists in the way an object is differentiated from other objects and consists in the way it is determined (1738, pp. 96–97). Finally, since objects sharing an essence belong to a species, and other objects belonging to a different species have a different essence, genera consist again in the similarity between essences of different objects (1738, p. 99). For example, windows and doors share the similarity that they are openings in the wall and therefore can be counted among a common genus (1738, p. 100). Insofar as Wolff argues that objects sharing an essence belong to a single species, he must have taken classifications of species and genera, if properly conducted, to reflect the essence of objects. Hence, Wolff’s essentialism is evident from his account of essence, genus, and species.

Wolff’s construal of essences no doubt differs from present day accounts of essences. Contemporary philosophers writing about essences and natural kinds often have a very restricted account of essence. They point to a limited set of examples such as chemical elements and fundamental particles as having essences and as being natural kinds. By contrast, Wolff basically takes the *structure* (mode of composition) of any composite object (eye, clocks, geometrical figures, etc.) to constitute its essence. However, although there are differences between Wolff’s essentialism and contemporary essentialism, Wolff shares the basic modal characterization of an essence as something that an object must necessarily have (Robertson & Atkins 2020). In addition, as we have seen, he is committed to the essentialist view, adopted by historical figures and contemporary philosophers and scientists alike, that essentialist kinds are “classes whose members share an essence from which their defining feature arises” (Kendler et al., 2011, p. 1143). This follows from Wolff’s Aristotelian method of logical division, in which attributes of an object follow from the essence of an object. Finally, Wolff’s account of real definition suggests that he also adopted the essentialist viewpoint that if an object has an essence we must be able to provide an account of how this object came about, i.e., we must be able to specify its cause. In the following, we will see that Sauvages was also committed to these viewpoints.

### Sauvages’ nosology

In this section, I will describe Sauvages’ nosology. In the first subsection, I will first analyze Sauvages’ philosophy of medical classification, as outlined in the initial discourse to his *Nosologie Méthodique* (1772). I argue that Sauvages’ adopts the logic of Wolff to provide classifications in medicine, and that he shares the essentialism of Wolff. In the second subsection, I analyze Sauvages’ views on psychiatric nosology. It will be argued that Sauvages’ again followed Wolff and tried to give real definitions of psychiatric diseases.

#### Sauvages’ philosophy of classification

In the initial discourse to the *Nosologie Méthodique* (1772), a translation of the *Nosologia methodica* (1763), Sauvages writes on the method of nosology. This method is once again greatly influenced by Wolff. Sauvages argues that in nosology we should adopt the so-called systematic method, which he describes as follows:

The systematic method groups together the diseases that resemble each other, and separates them from those that do not have a resemblance; it reduces all the individual diseases to their species, these species to their genera, the genera to orders, and these to a small number of classes. (2015 [1772], p. 489)

According to Sauvages, who here follows Wolff, we distinguish objects and diseases in terms of signs. Through signs, we achieve the aim of nosology, which is to distinguish the diseases from each other. Hence, if we want to cultivate nosology, we must know the signs of diseases. The botanists gave these signs the name of *Characters* (2015 [1772], p. 489). We enumerate signs through definitions, as was also argued by Wolff. Sauvages clearly follows Wolff’s account of definitions:

The *Definition* (la Définition) is the enumeration of the signs necessary and sufficient to make the defined object known, and to distinguish it from others. *Wolf. Logic 153*. It provides a complete and determined notion of the respective term. Therefore, in order to have a complete and established idea of a disease, it is necessary to define it or enumerate its proper signs and characters. (2015 [1772], p. 489)

We define diseases through providing the genus and the specific difference, which allow us to distinguish diseases from each other (2015 [1772], p. 490). In his commentary on Sauvages, King notes that in his early work Sauvages aimed to classify symptoms. Hence, it is symptoms that function as signs to differentiate diseases from each other. These symptoms, according to King’s reading of Sauvages, must be phenomena that are manifest, essential, and constant. The phenomena must be manifest to the senses, essential as opposed to accidental, and constant and invariable (King, 1966, p. 46).

In order to elucidate how nosologists should classify diseases, Sauvages quoted Sydenham, the great seventeenth-century English authority on medicine. He followed Sydenham in arguing that we should give a history or description of diseases and to establish a method of cure. In giving a history of diseases, we should “order them under defined and certain species, with the same care and the same exactitude as practised by the Botanists” (2015 [1772], p. 486). The goal was to discover the *essence* of diseases, without resorting to unfounded hypotheses concerning the disease (2015 [1772], p. 486). Thus, Sauvages quotes Sydenham as follows:

Similarly, it is not enough to observe the general symptoms of a disease that includes a variety of species. It is true that we do not notice the same variety in all the diseases, but there are several that authors arrange under the same class, without distinguishing their species, which differ between them in essence, as we shall see in what follows. There is more: in the case where one arranges the diseases according to their species, this is done relative to a hypothesis which replaced the truth of phenomena, so that this distinction is much less founded on the true character of the disease than on the hypothesis adopted by the Author. (2015 [1772], p. 486)

Hence, classification of diseases in terms of different species reflect the true essential nature of diseases. This view, shared by Sydenham and Sauvages, suggests that Sauvages was an essentialist concerning nosology. Martin (1990), discussing Sauvages’ *Nouvelles Classes des Maladies*, also stresses that Sauvages aimed to characterize the essence of diseases “Yet Sauvages was not only presenting a History–the careful description of observed symptoms of diseases–but, following Tournefort, he was presenting as well the essential characteristics of species of diseases” (1990, pp. 125–126).

In classifying diseases, we should not, according to Sauvages, rely on unfounded philosophical hypotheses (2015 [1772], p. 487). We should also classify diseases in terms of the *necessary symptoms* that accompany it. Sauvages again quotes Sydenham:

In third place in order to describe a disease, the symptoms that necessarily accompany it, and that are its own, are to be carefully separated from those that are accidental and fortuitous, such as those that depend on the temperament and age of the sick, and on the curative method that is employed; because it often happens that the disease varies according to the method being used, and the symptoms are much less the effect of the disease than the conduct of the Physician [...]. (2015 [1772], p. 487)

Sauvages described this method as entailing that one should describe the characteristics of symptoms that the disease constantly presents (2015 [1772], p. 487). The view that we should classify diseases in terms of their necessary symptoms mirrors the theory of logical division, described earlier.

Munsche and Whitaker (2012) argue that Sauvages classified diseases on the basis of symptoms, although they note his approach hinted at dissatisfaction with a purely symptom-based approach (2012, p. 228). King (1966, pp. 47–48), commenting on the *Pathologia methodica*, notes that for Sauvages pathology embraces both the study of phenomena and the study of causes. According to King, the study of causes is called etiology, while the study of phenomena comprises nosology. Williams (2003, p. 92) argues that for Sauvages “medicine must proceed only on the basis of what could be known directly, a principle that by definition excluded the search for causes […]”. I wish to argue that in his *Nosologie Méthodique*, Sauvages constructed a nosology that was based both on the study of symptoms and the study of causes. This is already evident from the initial discourse, in which Sauvages argues that a sure *theory* in medicine requires knowledge of physics and geometry, which supply knowledge of causes. Reflecting once again on the hierarchy of sciences, which we have discussed above, Sauvages notes:

I argue that only the study of Anatomy, experimental Physics and Mathematics can provide a sure theory; and as most of the Physicians ignore these sciences, it is not surprising that Aetiology is full of errors; an erroneous Aetiology is not useful to the Physician, like music to an architect, Aetiology will be unable to direct him in practice, or enhance the study of symptoms, for observation and experience, although most Physicians pretend to the contrary. (2015 [1772], p. 485)

Note that Sauvages argues explicitly that etiology should direct the practice of physicians, and that it can enhance the study of symptoms, on which we base our nosology. Hence, it seems that knowledge of causes is of direct relevance to nosology. The importance of studying causes for nosology has also been stressed by Martin (1990), who notes that Sauvages distinguished, like Wolff we may add (Martin is silent on the influence of Wolff on Sauvages), between historical (empirical or descriptive) knowledge, philosophical knowledge, which provides reasons for *why* certain phenomena occur, and mathematical knowledge, which provides knowledge of quantity. Sauvages describes the difference between historical, philosophical, and mathematical knowledge as follows:

We have only three ways to instruct ourselves and to extend our knowledge: namely, through History, Philosophy, and Mathematics. History is the knowledge of facts: for example, it teaches us that Pleurisy is accompanied by fever, breathing difficulties, cough, and chest pain. Philosophy is the knowledge of the causes and the principles; hence there is a philosophical knowledge of Pleurisy, which tells about the causes and principles of the four symptoms that accompany it, which are, for example, that they come from the inflammation of the pleura or the lungs. Mathematical knowledge consists in knowing the quantities and to know how to measure them; for example, to determine the strength and speed of the pulse, the degree of heat, the intensity of pain, the violence of the cough, and such other symptoms. (2015 [1772], pp. 487-488)

Sauvages, Martin argues, takes nosology to combine both historical knowledge and philosophical knowledge (1990, p. 135). Hence, nosology includes cognition of the *causes* of diseases. According to Martin, knowledge of causes was for Sauvages inextricably bound up with knowledge of the essence of diseases: “By knowing something of the essence of a species of disease, Sauvages believed he knew something about its *cause* as well […]” (Martin, 1990, p. 126). Hence, it seems clear that Sauvages was an essentialist about diseases, and that he thought, like Wolff, that knowledge of the essence of an object (such as a disease) requires knowing how this object comes about. In the next section, we will see that this viewpoint was also adopted within the nosological practice of Sauvages, insofar as he often cites causes in giving definitions of diseases and appends sections on *theory*, discussing the causes of diseases, to his classifications.

#### Sauvages and the practice of psychiatric nosology

In the psychiatric nosology of the *Nosologie Méthodique* (1772), Sauvages started with specifying the *character*, i.e., the definition and differentiating feature, of the class of vesaniae, i.e., illnesses that trouble or cloud reason (1772, p. 1). The character of vesaniae is that they are diseases of the soul. They consist in a “depravity of the imagination, of the appetite or of the judgment, or in a hallucination, a *bizarrie* or a delirium” (1772, p. 1). After defining the class, Sauvages defines the order of hallucinations, which are defined *causally* as errors of the soul, caused or occasioned by a vice or defect of the organs situated outside the brain (1772, p. 1). He then lists different genera of diseases belonging to this order, such as vertigo, suffusio, hypochondria, etc. (see for an overview Munsche and Whitaker 2012). Later in the book Sauvages gives an extensive treatment of these genera, listing their respective species, such as *Hypochondriasis biliosa* and *Hypochondriasis sanguinea* (1772, pp. 169–170). After dealing with hallucinations, Sauvages defines the order of *Morisitates* or *Bizarries* as depraved desires or aversions, and again lists a number of genera of diseases such as pica, bulimia, nymphomania, etc. (1772, pp. 2–3). Species are later listed such as *Pica infantilis* (1772, p. 205). In contrast to the other orders, Sauvages does not provide a causal definition of the order of *Morisitates* or *Bizarries*, although he offers etiologies in later discussions. The order of deliria is causally defined as an alienation of the mind caused by a defect of the brain, and contains genera such as mania (madness, *Folie*) and melancholia (1772, p. 4). In his treatment of these genera, Sauvages then lists the species, such as *Mania à pathemate*, mania caused by a passion, and *Mania ab hemicrania*, mania caused by a migraine (1772, pp. 393–397). In this way, Sauvages gave a hierarchical classification of mental disorders adopting the methods of the botanists.

Note that while defining psychiatric disorders, Sauvages often explicitly included the cause of these psychiatric disorders. In the terminology of Wolff, we can thus say that Sauvages did not merely provide nominal definitions of psychiatric disorders, but he also wanted to provide real definitions of these disorders, illuminating how these disorders come about. For Wolff, providing a real definition of an object meant explicating its essence. Given Sauvages’ essentialism, which we described in the previous section, it is likely that Sauvages also saw his causal definitions as explicating the essence of disorders or diseases.

Sauvages provides further insights into the cause of mental disorder in the section of *theory* on mental disorders. We have already seen that he attributed some mental disorders, such as deliria, including mania and melancholy, to a defect of the brain. However, in the section on theory Sauvages makes clear that the brain is not the sole cause of mental disorders. He argues, as Huneman has already noted (2008, p. 624), that mental disorders also arise from a mistake and the wrong use of our faculties. According to Sauvages, “The mistake stems not only from a bodily flaw […], but also from our own contempt for our faculties, and our lack of care in searching for the truth or cultivating our judgment” (Sauvages, 1772, p. 14. Translated and quoted by Huneman, 2008, p. 624). Hence, as Huneman concludes, Sauvages relates madness to a particular kind of moral vice. According to Sauvages, the more imperfect a man is, the more he resembles a beast, the more he neglects the cultivation of reason, and the more chance there is for developing mental disorders (1772, p. 11). Our insanity comes from the fact that we do not know how to curb our passions, and that we do not cultivate our faculties and judgment (1772, p. 12). Sauvages illustrates his views by noting that a peasant who suffers from cataract suffers from hallucinations, whereas a philosopher, who supposedly cultivates her judgment, recognizes the mistake and gets rid of it (1772, p. 14). He also states that although the majority of maniacs owe their disorder to a defect of the brain, there are some who owe their illness also to a vice of the soul (1772, p. 17). All of these remarks are meant to argue against materialists. If we adopt purely physiological or anatomical explanations of mental disorder, we are led to materialism and Spinozism, and If we adopt such a position, Sauvages argues, there would be no genuine responsibility and no moral philosophy (1772, pp. 13–14).

A core feature of Sauvages etiology of psychiatric nosology is that, according to him, the cause of such disorders can be both mental and physical. Thus, for example, next to a damaged brain, many people fall into madness because they are excessively occupied with some object (1772, p. 19). Huneman (2008) notes that this feature of Sauvage’s thought was a feature of eighteenth-century Montpellier vitalism. As Huneman describes (2008, p. 615): “Vitalism conceived of organisms as animal economies understandable through the transformations of the various modes of their sensibility. This allowed some physicians to define a kind of anthropological program, which viewed human beings as a whole, with no distinction between *le physique* and *le moral*”. According to Huneman, Sauvages argued that both physical causes and moral causes can generate mental disorders. As Huneman explains:

Here is the reason why, in mania, moral causes *and* physical alterations are both at work: while (1) the sympathy between brain and other centers of the “economy” explains the production of psychical symptoms, conversely (2) the moral affections clearly are possible causes of diseased organs. (Huneman, 2008, p. 625)

Hence, Sauvages had a complex conception of the etiology of mental disorders. However, he did not doubt that the cause of mental disorders could be clearly established and he referred to such causes in his definitions of mental diseases. As I have argued, understood from a Wolffian point of view, this amounted to providing real definitions of mental disorders, which explain their essence. If I am correct, it was partly because de Sauvages thought that we can establish the causes of mental disorders, that he adopted an essentialist psychiatric nosology.

We can conclude this section by wondering how Sauvages could argue that mental disorders have both physical and moral causes and still be an essentialist about mental disorders. For some present-day accounts of essentialism assume that mental disorders have a single cause (Kendler et al., 2011). Although my account must necessarily be a bit speculative, I will argue that the Wolffian conception of causation allowed for attributing a single complex cause to mental disorders while treating both physical causes (e.g., brain defects) and moral causes (e.g., being obsessed with something) as *partial causes* of mental disorders. We have already seen that King had established that Sauvages followed Wolff’s distinction between *principium* and *causa* (1966, pp. 48–49). Hence, Sauvages was aware of the Wolffian conception of cause. Importantly, Wolffians distinguished between complete causes and partial causes. Thus, for example, the Wolffian Baumgarten, who wrote a Wolffian textbook on metaphysics that was highly influential in the eighteenth century, 1argued, first analyzing the concept of a ground, that a sufficient ground is the complex of partial grounds that explain why something is the case, whereas an insufficient ground is merely a partial ground (Baumgarten, 1766, p. 8). Thus, for example, my having eaten lots of fast food in the last year is a partial ground for gaining weight, whereas this fact taken together with my metabolism, my exercise regime, my other nutritional food habits, and possible other factors is the sufficient ground of gaining weight. Now, causes are defined in terms of the concept of ground. More specifically, that which contains the ground of the actuality of something, i.e., that which explains why something is actual, is a cause (ibid., p. 83). Insofar as the concept of cause is understood in terms of the concept of a ground, we may expect that we can also distinguish between complete causes and partial causes of a thing. Indeed, Baumgarten appears to make this distinction when he argues that multiple causes of a caused thing are *Mitursachen* (*concausae*) who *come together* in order to cause a certain thing (Baumgarten, 1766, p. 85). Hence, complex phenomena can have a cause that is analyzable into multiple partial causes. In this way, we can argue that mental disorders have complex single causes, which are analyzable into, e.g., partial causes as brain defects and moral partial causes.

## Cullen’s nosology

Cullen (1710–1790) was chair of medicine at the University of Edinburgh. He was an internationally well-known scholar. In addition, he was an active medical practitioner himself, operating a blossoming consultation practice (Risse, 1974). Cullen devoted much of his time to writing a nosology that would allow physicians to correctly diagnose diseases (Munsche & Whitakker 2012). He was significantly influenced by Linnaeus and Sauvages, and in turn influenced many of his successors, such as Benjamin Rush, one of the founding fathers of the United States and one of the founders of American psychiatry, and the influential American physician Thomas Parke (Bell, 1950).

This section discusses, first, Cullen’s views on the methodology of nosology (4.1) and, second, his account of the causes of psychiatric disorders (4.2). I argue that Cullen’s Nosology by and large followed the method of classification as discussed in the section on essentialism and that his views on the methodology on classification show great similarity with that of Sauvages. I then describe Cullen’s views on the causes of psychiatric disorders, and argue that Cullen adopted the essentialist viewpoint that a single essential cause is the reason for the symptoms associated with psychiatric disorders.

### Cullen’s philosophy of classification

A nice guide to Cullen’s philosophy of classification can be found in his *Lectures Introductory to the Practice of Physic*, which comprise lectures of Cullen first published in 1827 and printed from copies of these lectures (1827a,1827b, I, p. v-vi). There, Cullen distinguishes between medicine (*physic*) based on an empirical plan, where we are guided by experience alone, and medicine based on a dogmatic plan, where we have recourse to reasoning and try to explain medical phenomena through their causes (1827a,1827b, I, p. 415). Cullen argues that although experience is indispensable in physics, we must always rely on a dogmatic plan to perfect medicine. He states that in medicine in particular and humanity in general there is a strong propensity to seek for causes of phenomena, and accordingly dogmatic reasoning in medicine is unavoidable (1827a,1827b, I, p. 417). Hence, Cullen concludes that it “is evident that reasoning, and what is called theory in physic, is unavoidable […]” (1827a,1827b, I, p. 419). This is also true for nosology, where we should strive to find the inner cause of external phenomena through dissection, which aims to find the proximate cause of diseases (1827a,1827b, I, p. 429):

It is, I think, now agreed, that the dissection of morbid bodies is one of the best means of improving us in the distinction of diseases. Sauvages indeed has rejected the employment of the internal seat of diseases as a means of distinguishing them; but he has, in an hundred instances, tacitly employed it; and under the ambiguity that often occurs in external symptoms, it is evident that dissection, by showing the parts singly or jointly affected, shows the real and steady changes in the system, upon which the external symptoms depend, and therefore must lead to the proper limiting of genera and species. (1827a,1827b, I, p. 423)

Here, Cullen argues, similar to Sauvages, that causes can be taken to individuate and identify diseases, insofar as the external phenomena by which we classify diseases in nosology are taken to result from an inner cause. This internal cause explains these phenomena and explains why the external phenomena co-occur. In line with this reasoning, Cullen argues that nosology is intimately connected to the study of causes of diseases in sciences such as pathology and physiology:

On the present subject, I think it must now appear evident, that the distinction of diseases must be often guided by the dissection of morbid bodies – must be constantly guided by anatomy, physiology, and pathology united together; and therefore, that the discernment and accurate distinction of external symptoms will be most effectually obtained by the cultivation of a Dogmatic system. (1827a,1827b, I, p. 424)

The dogmatic search for causes is thus of great utility for nosology, insofar as it is through causes that we can identify diseases and establish, in Cullen’s terms, the common nature of diseases (1827a,1827b, I, p. 435). As Cullen puts the point: “and even where these [organic affections] are in the internal parts, anatomy has often explained their connexion with external symptoms, so as to establish a common nature in different diseases more certainly than any observation of the symptoms alone” (1827a,1827b, I, p. 435). Hence, Cullen thinks that a common cause can account for all the external symptoms of a disease, and is thus a primary means to identify the nature of diseases with. Schematically, we can, drawing on Kendler and colleagues’ (2011) picture of essentialism, present this view as follows:
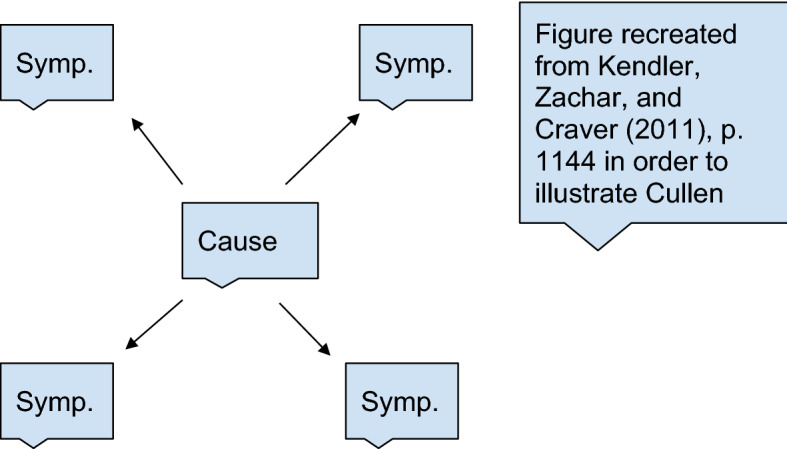


This is an essentialist picture of diseases because different symptoms are taken to be explained by a single underlying essentialist cause (although there are surely more varieties of essentialism). In the same way as the properties of gold are taken to follow from its essence, i.e., its atomic number, the symptoms of diseases are taken to follow from their cause (Kendler et al., 2011, p. 1144). Insofar as Cullen believes that the proximate cause of diseases accounts for all its symptoms, and we have seen there is evidence he adopts this picture, he can be taken to adopt this essentialist scheme.

In line with the importance he assigns to the study of causes for medicine, Cullen describes his general method in medicine as follows (1827a, 1827b, I, pp. 440–443). First one provides a history of a disease, i.e., an empirical description of all the symptoms that accompany a disease. Then, secondly, one investigates the proximate cause of the disease. Thirdly, one moves to nosology: from “the phenomena of the disease, and with a view to the conclusion respecting the proximate cause, I am next to enter into a critical disquisition with regard to the proper character and limits of every genus, and its division into species and varieties” (1827a, 1827b, I, p. 442). Fourth, one studies the remote causes of the disease, moves to the prognostic, and finally one studies the method of cure.

The focus on individuating and identifying diseases in terms of their causes is also evident in Cullen’s *Nosology* (1800 [1769]). There, Cullen argues that diseases are of the same kind or species if they arise from the same cause:

The one is, that similitude in the cause of the disease, argues a similitude in the disease thence arising: thus, when the diseases of two different persons arise from one and the same cause; when that cause is essential to the production of the disease in both; and when the same cause appears to be of the same quality, we may safely infer that such diseases are of the same, or of a similar kind. (Cullen, 1800 [1769], p. xv)

Next to individuating and identifying diseases through their cause, Cullen also argues that the similarity of diseases in different persons may be shown if there is a similarity in the remedies by which they are cured. In other words, we view diseases as being the same if they are cured by the same remedy (1800 [1769], p. xvi).

Interestingly, and not properly analyzed in the literature, Cullen wavers in his assessment of whether causes should be used as characters to define diseases. He does not reject his previously formulated account that species of diseases are identified in terms of causes. Cullen adopts this viewpoint throughout his work. However, the question is whether the characters which we use to describe diseases in nosology should only consist in external observable characters or symptoms or if we may include causes as well. In his lectures, Cullen argues that the characters used to define diseases should be observable symptoms, since there is much disagreement between medics on the cause of diseases:

The *fourth* rule is, that the characters should be absolutely free and independent of all theory and hypothesis. Sauvages, in his Prolegomena, mentions ten or twelve definitions of Pleurisy, all taken from some view of the proximate cause; but all of them would now be entirely rejected. By looking into the systems, however, you will perceive that physicians have gone on in the same track of defining diseases by their proximate causes, which are in many cases disputable, and may long be so. (1827a, 1827b, I, p. 457)

Cullen is quick to note that this only concerns the definition of diseases given in nosology, and that he retains the idea that species of diseases are identified by causes. Thus he states that he has previously said that the internal seat is used to identify the cause of diseases, but that he should be read as saying that the internal seat belongs to the history of disease, and is thus still relevant for nosology, but that it should not be used as a character in the definition of a disease, which should consist of externally observable symptoms (1827a, 1827b, I, p. 458). Interestingly, Cullen was not consistent in his views. In his *Nosology*, Cullen maintains that the cause can be used in the definition of a disease as a character if it is well known:

Ought the cause of a disease to make any part of the definition?

To this it may be answered, that as the judgment formed by physicians of the causes of diseases, is often fallacious, and even false, and therefore not to be rashly relied on in distinguishing diseases; yet, as these causes are sometimes sufficiently certain, and easily to be observed, they may be admitted in Nosology, as legitimate characters. (1800 [1769], p. xviii)

The logic Cullen employed in his nosology can be described as the traditional logic of classification, expounded in the section on essentialism and still articulated by Wolff and other logicians in the eighteenth century. Cullen notes that “all diseases, in order to be easily and certainly discriminated, should be arranged, like systems of Botany, by genera and species, with characteristic definitions: that is, by a methodological Nosology” (1800 [1769], p. v). In the denomination of diseases, Cullen followed Linnaeus, arguing that his rules for naming classes are taken from Linnaeus in the Critica and Philosophica Botanica (1800, p. xxi).

Species of diseases should be characterized, according to Cullen, by essential and necessary characters, which further illustrates his essentialism. In his *Nosology*, Cullen chides classifications which detail symptoms that seldom attend the disease, as opposed to those that are *necessarily connected* with it, common, and inseparable (1800 [1769], p. iv). In his lectures, Cullen notes that we must take pains to “distinguish between what are the essential and what the accidental symptoms.” (1827a, 1827bb, I, p. 447). Hence, Cullen distinguished between necessary and essential symptoms of a disease, in terms of which we must define a disease, and the contingent accidental symptoms.

### Cullen and the cause of psychiatric disorders

Cullen construed an order of vesaniae, or impaired judgment, within the class of neuroses. This order contained diseases such as amentia, or imbecility of the judgment, melancholia, or partial insanity, and mania, or universal insanity (1800 [1769], pp. 130–133). After specifying genera, he would treat the different species of disease. In this way, Cullen classified the vesaniae in terms of genera and species.

In his *First Lines of the Practice of Physic* (1784), Cullen treats the cause of the vesaniae, i.e., the disorders of the intellectual functions. He starts off by discussing delirium, which consists, according to Cullen, in erroneous judgment (1827a, 1827b [1784], II, p.510). A delirium is defined as a false or mistaken judgment of relations of things, about which most men form the same judgment. In addition, delirious persons form judgments that are very different from the judgments that the person had formed before (1827a, 1827b [1784], II, p.510). This false judgment is frequently associated with a false perception of external objects, or a very unusual association of ideas, or a disproportionate emotion or passion (1827b [1784], II, pp. 511–512). This leads to the following definition of delirium:

Delirium, then, may be more shortly defined, -In a person awake, a false judgment, arising from perceptions of imagination, or from false recollection, and commonly producing disproportionate emotions. ((1827a, 1827b [1784], II, p.512)

Insanity is defined as a particular kind of delirium, one without pyrexia and comatose affection, and Cullen sets out to find the cause of delirium in general. He argues that the connection between body and mind is such that delirium must have a corporeal cause (1827a, 1827b [1784], II, pp.512–513). The part of the body connected with the functioning of the mind is the brain (1827a, 1827b [1784], II, p.513). According to Cullen, it is probable that the state of the intellectual functions depends on the nervous power, a subtle fluid present in every part of the medullary substance of the brain and nerves (1827b [1784], II, pp. 513–514. For further discussion of this nervous power, see Jackson, 1983, p. 311). This nervous power can be in a state of mobility and force that is sufficient for the exercise of the intellectual functions, which is called *excitement*, or it can be in a state that is not sufficient for the exercise of the functions, which is called *collapse* (1827a, 1827b [1784], II, p. 514). These states of excitement and collapse correspond to states of waking (excitement) and sleeping (collapse) (1827a,^1827^b [1784], II, p. 515). The change from collapse to excitement, as witnessed for example when moving from sleeping to waking, is one of degrees. From this Cullen concludes “that not only the different states of excitement and collapse can take place in different degrees, but that they can take place in different parts of the brain, or at least with respect to the different functions, in different degrees.” (1827a, 1827b [1784], II, pp. 515–16). In the transitions from waking to sleeping and from sleeping to waking, i.e., in “intermediate state of unequal excitement”, we witness delirium, false perceptions, false associations, and so forth (1827a, 1827b [1784], II, p. 516). This shows, according to Cullen, that delirium depends “upon some inequality in the excitement of the brain” (1827a, 1827b [1784], II, p. 516) This is further proven by the fact that in dreams we witness delirium and that in case of fever, i.e., a case of unequal excitement of the brain, patients also often suffer from delirium (1827a, 1827b [1784], II, pp. 516–517). Hence, Cullen concludes that delirium “may be, and frequently is, occasioned by an inequality in the excitement of the brain (1827a, 1827b [1784], II, p. 517). On this basis, Cullen concludes that insanity is the result of different states of excitement of the brain (1827a,1827b [1784], II, p. 519). In line with his account of the cause of insanity, Cullen reduces all symptoms of psychiatric disorders to this cause. Thus, for example, mania is characterized sometimes by a false perception or imagination, a false judgment concerning a single object, and a mind that rambles from one subject to another or hurry of the mind, among others ((1827a, 1827b [1784], II, p. 521). All such symptoms are explained as follows:

It appears to me, that the whole of these circumstances and symptoms point out a considerable and unusual excess in the excitement of the brain, especially with respect to the animal functions [...]. (1827a, 1827b [1784], II, p. 522)

Here we see, once again, how Cullen adopts an essentialist account of psychiatric disorders. Such disorders are characterized by a multiplicity of symptoms, but all of these symptoms are explained in terms of a single essential cause, which is responsible for the multiple observable symptoms.

## Conclusion

In present-day psychiatry and history of psychiatry, debate about the ontology of mental disorders, and in particular on the question of whether mental disorders are natural kinds, is prevalent. However, historians of psychiatry who have dealt with questions dealing with essentialism, natural kinds, and nosology, have paid little to no attention to the impact of the sciences of logic and metaphysics on conceptions of medical and psychiatric method, the natural of mental disorders, and the classification of mental disorders. Historically, however, logic and metaphysics have significantly shaped methods and interpretations of classification in the natural sciences. This paper corrects this lacuna in the history of psychiatry by analyzing the impact of Christian Wolff’s logical and metaphysical theories on the conception of medical method and (psychiatric) nosology of Boissier De Sauvages. Wolff accepted the Aristotelian method of logical division and adopted the view that species pick out essences of objects. Wolff exerted a significant impact on Sauvages, who, as I have shown, adopted Wolff’s conception of science, his views on logic, in particular his views on definitions, and also adopted the theory of division. In my view, we can posit considerable continuity between Wolff’s views and the nosological practice of Sauvages, much more then has so far been recognized in the literature. I have argued that Sauvages attempted, in line with Wolff, to provide real definitions of (psychiatric) disorders, i.e., definitions which explicate how disorders come about and that explicate their essence. This would explain why Sauvages stressed the importance of giving causal definitions of (psychiatric) disorders, even if some commentators have interpreted Sauvages’ nosology as a purely symptom-based approach to classification. There is considerable continuity between the methods of Sauvages and those of William Cullen, much more then has so far been recognized. Cullen adopted an approach to nosology according to which we identify and individuate a species of disease by locating its proximate cause. This led him to explain the multiple observable symptoms of psychiatric disorders in terms of a single essential cause, which demonstrates his essentialism. Hence, the concept of a mental disorder adopted by influential eighteenth-century nosologists was that (i) psychiatric disorders are similar to other medical disorders in having an essence, (ii) from this essence it followed that psychiatric disorders had necessary characteristics (attributes) in terms of which we can describe psychiatric disorders, (iii) we can classify psychiatric disorders in species and genera and provide classifications of genera and species that carve nature at its joints. Future research should determine whether this essentialist perspective influenced nineteenth and twentieth-century nosologists and when the essentialist view on mental disorders became an object of critique.

## Footnotes

1 Baumgarten closely followed the account of causation of Wolff. See van den Berg 2014, chapter 4.
